# Prevalence and Potential Impact of Gastrointestinal Insufflation During Cardiopulmonary Resuscitation

**DOI:** 10.3390/jcm14072511

**Published:** 2025-04-07

**Authors:** Maximilian Andreas Fichtl, Sophia Anna Henne, Viktoria Bogner-Flatz, Michael Dommasch, Philipp Zehnder, Karl Georg Kanz, Wilhelm Flatz

**Affiliations:** 1 Department of Orthopedics and Trauma Surgery, Musculoskeletal University Center Munich (MUM), University Hospital, LMU Munich, Ziemssen Str. 5, 80336 Munich, Germanyviktoria.bogner-flatz@aelrd-bayern.de (V.B.-F.); 2 Department of Anesthesiology, Intensive Care and Pain Medicine, Hospital Harlaching, The Munich Hospitals LTD, 81545 Munich, Germany; 3 EMS Authority of Munich, Ruppertstraße 19, 80337 Munich, Germany; 4 TUM School of Medicine and Health, Emergency Department, University Medical Center, Technical University of Munich, 81675 Munich, Germany; 5 TUM School of Medicine and Health, Department of Trauma Surgery, University Medical Center, Technical University of Munich, 81675 Munich, Germany; 6 Department of Radiology, University Hospitals, Ludwig Maximilian University of Munich (LMU), 80539 Munich, Germany; radiologie.muenchen@gmail.com

**Keywords:** resuscitation, OHCA, insufflation, aspiration

## Abstract

**Background/Objectives:** Insufflation of the gastrointestinal tract, as a side effect of improper ventilation, is a known complication in resuscitation patients. As animal studies have shown, this can be associated with an increase in intra-abdominal pressure with adverse effects on hemodynamics and respiratory mechanics. In this study, we investigated the prevalence and severity of insufflation and discussed the potential impact on the outcome of resuscitation. **Methods:** This study was based on computed tomography (CT) images from two university hospitals in Munich, Germany, which were taken as part of the trauma room care of out-of-hospital cardiac arrest (OHCA) patients. According to local resuscitation protocol, CT performed during ongoing cardiopulmonary resuscitation or after the return of spontaneous circulation (ROSC) was archived to determine the potentially reversible cause of cardiac arrest. CT images from 2014 to 2018 were analyzed in this study. Using an advanced visualization and analysis platform for medical image data, the gas volume within the gastrointestinal tract was determined and compared between resuscitations with lethal and secondary survival outcomes. **Results:** A total of 92.44% of included OHCA patients (*n* = 172) showed signs of increased gastrointestinal gas volume in comparison to the physiologically prevalent gas volume. In OHCA patients with a lethal outcome, significantly more gas was detected in the gastrointestinal tract with a median of 757.40 mL compared to 380.65 mL in resuscitations with secondary survival (*p* ≤ 0.05; W = 4278). Furthermore, Cohen’s r was used to calculate the effect size, indicating a weak association with the outcome of resuscitation (r = 0.24). In addition, a logistic regression analysis was performed to examine the influence of age, gender (female), and the gas volume of the intestines and stomach on the dependent variable “death”. The analysis shows that the model, as a whole, is significant (Chi^2^ = 17.67; *p* 0.02; *n* = 172) and supports the hypothesis that intestinal insufflation correlates with a lethal outcome from resuscitation (b = 0.001; OR 1.001 (95% CI [1.000–1.002]; *p* = 0.021). **Conclusions:** Insufflation in resuscitation patients is a common phenomenon with potential consequences for the outcome. Even if the effect we have shown appears small, the outcome of resuscitation patients can possibly be improved by preventing or correcting insufflation. To understand its potential impact on resuscitation outcomes fully, further work must be performed to investigate causality.

## 1. Introduction

Insufflation of the gastrointestinal tract is a known complication during cardiopulmonary resuscitation [1,2]. As a result of pulmonary ventilation, accidental insufflation of the stomach and consecutive accumulation of air might occur [3]. Various factors are known to favor the insufflation of the stomach. These include high tidal volumes, a short inspiratory time, and the use of high ventilation pressures [4,5] or ventilation of an unsecured airway during mouth-to-mouth or bag–mask ventilation [6]. In addition to these factors, there are other mechanisms, such as esophageal intubation, which directly cause insufflation of the gastrointestinal tract (GI-tract) [7].

Insufflation of the stomach can result in various adverse effects, with regurgitation and aspiration causing potential airway obstruction being the best-known complication [1]. In addition, various studies have shown that insufflation in cardiac arrest is associated with various adverse effects on hemodynamics and respiratory mechanics [8,9,10,11].

Particularly in the preclinical area and in emergency medicine, where the prevailing conditions mean that an airway cannot be secured quickly or a rescue can be lengthy and difficult, the airway and the associated complications are of particular importance.

To our knowledge, the prevalence and extent of possible insufflation of the GI-tract in humans in the context of a periarrest situation have been insufficiently investigated to date. The aim of this study was to determine the prevalence of insufflation in OHCA patients using CT images and investigate its potential impact on patient outcomes.

At the University Hospitals Rechts der Isar of the Technical University of Munich and at the campus of the City Centre Clinic of the Ludwig Maximilian University of Munich, the admission of non-traumatic OHCA patients is performed via the hospitals’ trauma room. Based on an established algorithm, a whole-body CT scan can be performed to exclude possible reversible triggers of cardiac arrest. Performing CT scans after and during cardiopulmonary resuscitation is technically possible and might favor the outcome in certain conditions, as shown by studies [12,13,14]. The prerequisite for CT scans during resuscitation while maintaining minimal no-flow time is that the team performing the procedure is familiar with the methodology and experienced in its use. In certain circumstances, the use of CT in resuscitation patients is recommended by the resuscitation guidelines [15].

This work is based on the CT scans acquired as part of the treatment of OHCA patients. Using an established visualization and analysis platform for medical image data, the gas volume in the GI-tract could be calculated and compared between resuscitations with secondary survival and lethal outcomes.

If insufflation of the gastrointestinal tract can be confirmed as an independent factor for the outcome of resuscitation, resuscitation guidelines should be used to define measures to prevent or promptly rectify insufflation.

## 2. Materials and Methods

For the retrospective analysis, all patient admissions to the trauma rooms of the two participating hospitals were searched for the admission diagnosis of “resuscitation”. For the period of 2014–2018, 361 patients were identified and further analyzed. In the second step, these patients’ records were checked to see whether a CT scan had been performed as part of the trauma room diagnostics. If the abdomen was fully recorded and there were no artifacts that could falsify the measurements, they were included in the study population. A total of 172 patients were identified.

The software package 3D-Slicer Version Slicer 4.11.20210226 (https://www.slicer.org/ (accessed on 25 January 2021)) [16], an advanced and established visualization and analysis platform for medical image data, was used for the volumetric analysis. Using a threshold-based segmentation function, all gas-filled areas of a CT image were first visualized. All areas corresponding to structures outside the GI-tract were manually removed. The gas volume was initially measured for the complete GI-tract from stomach to colon. Gas in the esophagus or sigmoid colon was not included in the calculation. The gastric gas volume was then measured separately so that the difference between the gastric gas volume and the GI-tract gas volume could be used to calculate the gas volume for the intestine.

**Figure 1:**
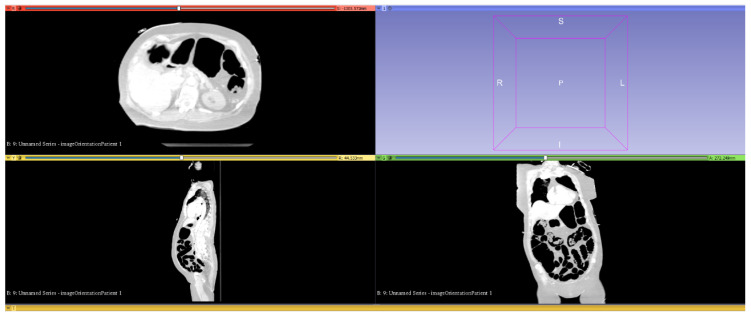
Uploaded whole-body CT scan with highlighted and segmented intestinal gas.

Figure 1 shows a whole-body CT scan of an OHCA patient that was loaded into the software. For manual processing, there are three correlating slice planes and a window for displaying the marked volume using the threshold function (top right).

**Figure 2:**
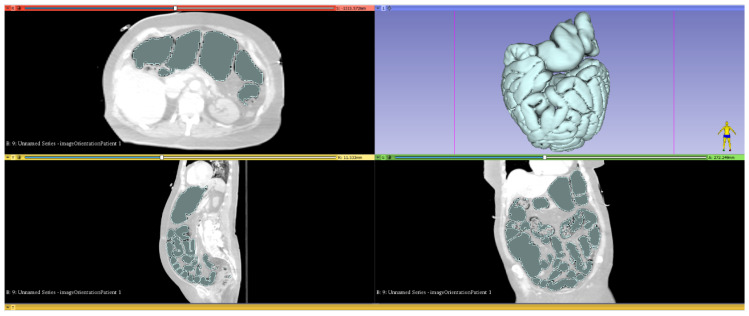
Visualization and segmentation of a CT scan utilizing analytic software.

Figure 2 shows the gas-filled areas within the MDT that were marked using the “threshold” function. This was preceded by the manual removal of marked areas that were located extracorporeally and thoracically.

**Figure 3:**
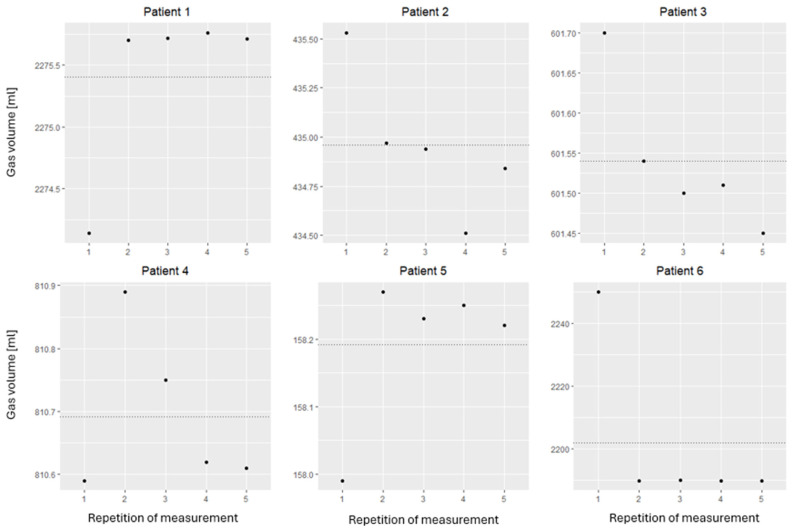
Remeasurement for internal validity.

To determine the internal measurement validity, the gas volumes were repeatedly measured using six randomly selected patients and comparing the measurement results (see Figure 3). For an objective judgment, the relative error of the measurement repetitions was determined. This was defined as the range between the maximum and minimum, divided by the mean value. This was at a maximum of 2.73% for patient Nr. 6 and <0.3% for the remaining patients. Given the minimal deviation in the individual results, good internal measurement validity can be assumed.

Figure 3 visualizes the repeated measurements used to assess the internal measurement validity. The horizontal axis represents the measurement repetition, and the vertical axis shows the volume in milliliters. The points describe the individual measurements of each repetition measured in milliliters. The horizontal dotted line represents the calculated mean value of the measured volumes.

## 3. Inclusion and Exclusion Criteria

All adult OHCA patients aged 18 and over who were admitted via the interdisciplinary trauma room were included, provided a complete CT scan of the abdomen was available. The patients for whom the CT scan range did not fully cover the abdomen, or in cases where the image quality was inadequate to perform reliable volumetric measurement, were excluded.

## 4. Grouping and Study Endpoints

The endpoints between the two comparison groups were “secondary survival” and “exitus letalis”. In the case of exitus, no distinction was made between death in the trauma room or later during hospital treatment. Additional patient demographic information was obtained using the hospitals’ internal IT systems.

## 5. Statistical Analysis

The volumetric results were given in ml and compared between the surviving and lethal resuscitation groups. Initially, the data were tested for a normal distribution using the Shapiro–Wilk test, which showed that they were not normally distributed. The Mann–Whitney U test (Wilcoxon rank sum test) was used to assess the correlation between intra-abdominal gas and outcome. In the case of statistical significance, Cohen’s d was used to determine the effect size. A *p*-value < 0.05 was defined as the significance level. The statistical analysis was performed using SPSS Version 28.0. (IBM Corp. IBM SPSS Statistics for Windows [Internet], Armonk, NY, USA: IBM Corp; 2017. URL https://www.ibm.com/de-de/analytics/spss-statistics-software (accessed on 12 December 2021)).

In addition, a logistic regression analysis was performed with the available demographic characteristics, the outcome, and the gas volume. Furthermore, a Receiver Operating Characteristics (ROC) analysis was performed.

## 6. Results

Of the 361 patients admitted, 172 patients underwent CT scanning as part of the emergency room treatment. Table 1 shows the age and outcome of all OHCA patients who were admitted to the trauma room of the participating hospitals during this period.

Table 2 shows the outcome of all admitted patients who underwent a CT scan.

The gas volumes for the entire gastrointestinal tract and for the stomach could be measured directly using the method described above. The gas volume of the intestine was calculated as the difference between the two compartments mentioned.

In the OHCA patients examined by CT (*n* = 172), a median of 636.46 mL of gas was detected in the GI-tract. In the case of a lethal outcome, the median absolute volume of 757.40 mL was significantly higher than the volume of 380.65 mL in the patients with secondary survival. A maximum gas volume of 5549.79 mL was detected in one of the patients. In comparison, the minimum measured volume was 86.32 mL.

For the stomach, a median of 100.85 mL was detected in the patients with successful resuscitation compared to 172.35 mL in the patients with lethal resuscitation.

For the intestine, a median of 394.68 mL was detected in the patients with successful resuscitation compared to 209.76 mL in the patients with lethal resuscitation.

Considering the large standard deviation, it can be concluded that there is a large variance between the patients examined and their gas volumes (Table 3).

The Wilcoxon–Mann–Whitney U test (Wilcoxon rank sum test) was used to assess the significance of the two trends (deceased vs. survived) and the volumetric results obtained. The null hypothesis states that there is no difference in the outcomes between the comparison groups. A value of 5% was set as the significance level.

An increased gas volume in the GI-tract correlates significantly (*p* ≤ 0.05) with a lethal outcome compared to resuscitation with secondary survival. The effect size is r = 0.24 and corresponds to a small effect according to Cohen.

Similarly, an increased gas volume in the intestine is associated with an increased probability of a lethal outcome (*p* ≤ 0.05). The effect size is r = 0.28 and corresponds to a weak effect according to Cohen.

An increased gas volume in the stomach does not correlate with the lethal outcome of resuscitation (*p* = 0.28).

In addition, a logistic regression analysis was performed to examine the influence of age, gender (female), and the gas volume of the intestines and stomach on the dependent variable “death”. The analysis shows that the model, as a whole, is significant (Chi^2^ = 17.67; *p* = 0.02; *n* = 172) but with poor variance explanation, according to Nagelkerker’s R^2^ = 0.10, in line with the recommendations of Backhaus et al. (2006) [17] (Table 4).

**Figure 4:**
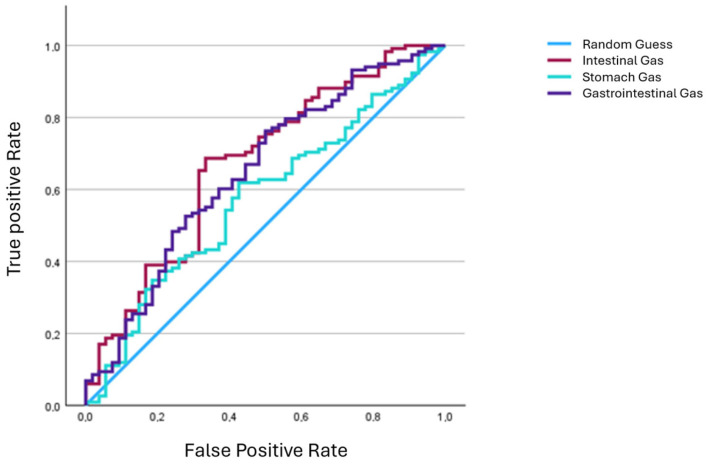
Receiver Operating Characteristic curves.

The ROC curve in Figure 4 shows the diagnostic accuracy of the possible factors intestinal gas, stomach gas and gastrointestinal gas for predicting death after resuscitation. The y-axis represents sensitivity (true positive rate), i.e., the probability that a model correctly recognizes a positive case. The x-axis represents specificity (false positive rate), i.e., the probability that model incorrectly classifies a negative case as positive.

The straight light blue line represents the reference line and describes a random classification. The other lines describe the predictive power of the examined variable intestinal gas, stomach gas and gastrointestinal gas. 

All curves lie above the reference line, indicating that the insufflated gas may be a predictor for the outcome of a resuscitation.

The fact that the curve for intestinal gas is above the other curves suggests that this may allow for better discrimination than the other variables.

The area under the curve (AUC) value of an ROC analysis describes the discriminatory power of a test, i.e., how effectively it can differentiate between positive and negative cases. An AUC value of 0.5 indicates a random classification with no diagnostic value. Values between 0.6 and 0.7 indicate moderate discriminatory power, and values > 0.7 indicate good discriminatory power (Table 5).

According to the ROC analysis, the gastric gas volume with a value of 0.574 is not a reliable predictor of a lethal outcome of resuscitation (*p* = 0.112).

The intestinal gas volume is statistically significant (*p* ≤ 0.05) with moderate predictive power in relation to the outcome of resuscitation. Moreover, the lower value for intestinal gas is well above 0.5, which further supports a possible significance of intestinal gas volume for resuscitation outcome.

## 7. Discussion

The aim of this study was to analyze OHCA patients regarding the prevalence and extent of possible insufflation. This was followed by a statistical analysis of the outcome of resuscitation and the volume of insufflation. While there are individual animal studies on this topic [8,9,10] and studies related to stomach insufflation [18], to the best of our knowledge, this study is the first that has performed volumetry of the entire GI-tract in a larger study population.

Gas is physiologically present in the GI-tract due to fermentation and aerophagy [19]. In previous studies, a physiological volume of 95 mL [20] or postprandial volumes of 149 ± 21 mL [21] and 220.6 mL in patients with functional gastrointestinal complaints were described [22]. Assuming a physiological volume of 149 ± 21 mL, an increased gas volume within the GI-tract was detected in 92.44% of the cases we examined (*n* = 172).

As our data show, there is a correlation between insufflation of the intestine and the increased probability of a lethal outcome during resuscitation. In a study by Naito et al. [18], the correlation between the insufflation of only the stomach and the outcome of resuscitation was investigated using a comparable study design. In addition to the calculated gas volume (400 vs. 417.59 mL), the results of their study also agree with ours in that insufflation of the stomach does not correlate with the outcome of resuscitation. However, Naito et al. conclude that insufflation of the intestine might show otherwise. Our study results indicate that increased insufflation of the intestine does indeed correlate with the increased probability of a lethal outcome in resuscitation patients.

**Figure 5:**
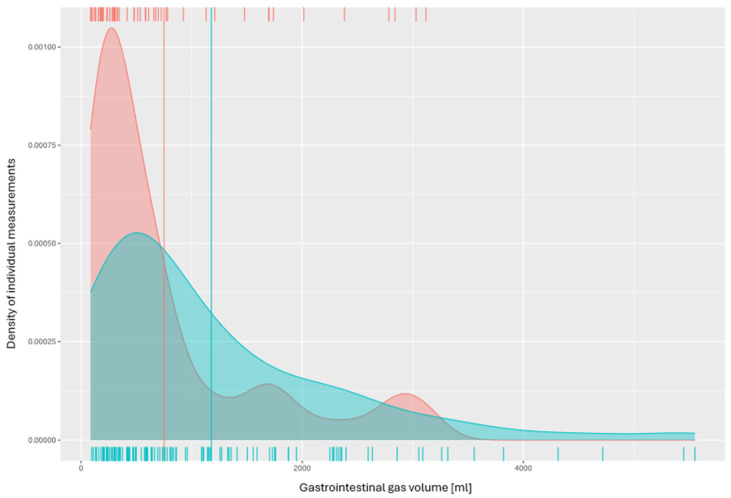
Outcome and gas volume of resuscitation patients.

Figure 5 visualizes the relationship between the gas volume determined in the GI-tract and the outcome. The horizontal axis describes the gas volume in milliliters, and the vertical axis describes the density of the individual measurements taken in relation to each other—it is unitless. The small red increments at the upper edge represent patients with secondary survival, and the small blue increments at the lower edge represent resuscitations with a lethal outcome. In areas with a high density of measured volumes, the small increments are closer together and the graph rises. Conversely, the sections in which the increments are further apart and the graph is flat show that a volume measurement produced a corresponding result less frequently. The two vertical, long lines show the median gas volume in the MDT for the deceased patients (blue line) and successful resuscitations (red line).

As can be seen in Figure 5, insufflation with small volumes predominates in the patients with secondary survival after resuscitation. The resuscitations with a lethal outcome show a higher incidence of insufflation with larger volumes.

In the logistic regression analysis we performed, a significant correlation between a lethal outcome of resuscitation and intestinal insufflation was demonstrated; however, this was only minimal, as measured by the odds ratio. We suspect, however, that in individual subgroups, especially in smaller patients and those with low BMI, the effect is significantly stronger.

There are various studies that have investigated possible mechanisms of insufflation in the context of cardiopulmonary resuscitation. The triggers described include ventilation of the unsecured airway with and without bag–mask ventilation [6,23] or the improper use of advanced airway devices [24].

As Paal and Braun et al. were able to show in various animal studies [8,9,10], the accumulation of air can lead to a consecutive increase in pressure in the abdomen, which is also transmitted to the thorax via the diaphragm. Among other things, the following pulmonary and hemodynamic effects of insufflation have been observed in animal experiments (cf. Table 6):

The extent to which these observations can be transferred to humans in the context of resuscitation is unclear. The fact that abdominal insufflation in humans has consequences for hemodynamics and respiratory mechanics is known and investigated in the context of capnoperitoneum for laparoscopy [25,26,27].

There are various factors related to ventilation that are known to promote gastric ventilation. In addition to esophageal intubation, bag–mask ventilation plays a particularly important role in this regard. During bag–mask ventilation, high tidal volumes, the use of high ventilation pressures, and a shortened inspiratory time can promote gastric ventilation.

If intestinal insufflation can be confirmed as a causal, independent factor affecting the outcome of resuscitation, measures should be taken to prevent insufflation during resuscitation. Possible options would be, for example, the early placement of a gastric tube or targeted training in correct ventilation.

In order to make conclusive recommendations in this regard, further studies must be conducted, in our opinion. In particular, the inclusion of patients’ BMI and the temporal aspects of preclinical resuscitation (e.g., duration of bag–mask ventilation, initial rhythm, or total duration of resuscitation) should be included in order to obtain comparable values.

## 8. Limitations

Various limitations must be considered when interpreting this study’s results. These include the following:

(1) Insufflation probably takes place as part of the pre-hospital resuscitation measures [28,29]. Accordingly, factors such as the duration of ventilation of an unsecured airway, the duration of the resuscitation, the change in the ventilation method, primary rhythm, and time of defibrillation would have been of particular importance. We suspect that prolonged resuscitation or prolonged ventilation of an unsecured airway is preceded by extensive insufflation. Given the time-critical nature of the operation, such documentation is often incomplete and inaccurate. It is therefore unfortunately not possible to analyze the written documentation of the ambulance service.
(2) Further patient characteristics such as height, weight, and body mass index might be of interest for a more in-depth interpretation of the data. We suspect that the potential consequences of insufflation increase the larger the volume and the smaller the person. Due to the retrospective study design, it was not possible for us to include these data. For an optimized statistical analysis, patient characteristics such as weight, height, and BMI should be included in further studies.
(3) As has been shown in animal experiments, the insufflation of gas leads to an increase in abdominal and thoracic pressure with associated adverse effects [8,9,10]. Whether these observations can be transferred to humans is unclear. A change in intra-abdominal pressure could not be demonstrated by our study design, even if the comparable pathomechanism in the context of surgical capnoperitoneum [25,26] seems conceivable.
(4) In accordance with the retrospective design of this study, a selection bias could not be completely ruled out. This study only included OHCA patients who were transported to the hospital under ongoing resuscitation or after ROSC was achieved. Patients who were pronounced dead at the scene could therefore not be included in this study. It is unclear how this possible bias affects the volumetry results.
(5) Based on this study, it is not possible to prove a causal chain between insufflated air, intra-abdominal pressure, and outcome. The poorer outcome in patients with increased gas volumes could also be explained by insufficient oxygenation because of insufficient ventilation or prolonged resuscitation attempts. In this case, the increased gas in the MDT would merely be a confounder.
(6) The gas volume was determined using a threshold function. The measurement range was defined as −500 to −1024 HU. By definition, air has an HU value of −1000 HU. As the air is humidified and voxels are sometimes a mix of solids and gas in the MDT, the measuring range for the volumetry must be adjusted accordingly. If the measuring range is larger, a larger volume is calculated. After reviewing various studies on this topic, we ultimately decided in favor of a threshold range from −500 to −1024 HU [22,30,31]. Whether this optimally represents the actual volume is unclear. Furthermore, to the best of our knowledge, there is no method that includes the expected compression of the gas due to the intra-abdominal pressure increase in the calculation.

## 9. Conclusions

The majority of OHCA patients showed a significantly increased gas volume within the GI-tract in comparison to the physiologically prevalent gas volume. For resuscitations with a lethal outcome, a significantly increased volume was found in the intestine compared to resuscitations with secondary survival. However, there was no correlation in relation to gastric gas volume.

The causal chain of the correlation between increased gas volume and exitus must be investigated in further studies. Our results suggest that an increased gas volume in the intestine correlates with an increased probability of lethal outcomes of resuscitation. However, it is unclear whether this is actually an independent factor. This study could not investigate whether the increased volume results in an increase in abdominal pressure with adverse effects on hemodynamics or whether it is merely a confounder of inadequate ventilation, insufficient oxygenation, or prolonged resuscitation.

The odds ratio of 1.001 that we calculated using logistic regression analysis suggests only a very small but significant effect. If insufflation can be confirmed as an independent factor in the outcome of resuscitation, resuscitation guidelines should be used to define recommendations for preventing insufflation or correcting it during resuscitation.

## Figures and Tables

**Table 1:** Outcome of all admitted OHCA patients.

	Patients		Age in Years		
*n* = 361	Male	Female	Median	Mean Value	Min/Max
total	255	106	67	64.2	18/99
Surviving	82	28	64	62.6	27/99
Deceased	173	78	69	64.9	18/97

**Table 2:** Outcome of admitted OHCA patients with CT scans.

	Patients		Age		
*n* = 172	Male	Female	Median	Mean Value	Min/Max
total	118	54	68	64.9	18/99
Surviving	41	13	66	62.9	29/99
Deceased	77	41	70	65.1	18/97

**Table 3:** Volumetric results in milliliters.

	Survived	Deceased	Total
Median Volume GI-Tract	380.65	757.40	636.46
Average Volume GI-Tract ± SD	750.53 ± 817.59	1178.05 ± 1133.18	1043.83 ± 1060.83
Max. Volume GI-Tract	3119.03	5549.79	5549.79
Min. Volume GI-Tract	86.32	96.38	86.32
Median Volume Stomach	100.85	172.35	148.42
Average Volume Stomach ± SD	342.78 ± 490.89	451.83 ± 500.04	417.59 ± 498.34
Max. Volume Stomach	1995.78	2113.31	2133.31
Min. Volume Stomach	0.00	0.00	0.00
Median Volume Intestine	209.76	394.68	341.25
Median Volume Intestine ± SD	407.74 ± 503.21	726.22 ± 838.27	626.23 ± 762.40
Max. Volume Intestine	2556.47	4724.13	4724.13
Min Volume Intestine	45.15	71.94	45.13

**Table 4:** Logistic regression analysis results.

					95% Confidence Interval	
	Regression Coefficient b	Standard Error	Sig.	OR	Lower Value	Upper Value
Age	0.015	0.010	0.135	1.015	0.995	1.034
Female	0.499	0.382	0.191	1.647	0.779	3.481
Gas volume intestine	0.001	0.000	0.021	1.001	1.000	1.002

1. Age: Age shows a positive, non-significant influence on the probability of death (b = 0.015; OR 1.015; *p* = 0.135). 2. Gender (female): in female patients, the odds of death are increased compared to male patients, but this effect was not significant (b = 0.499; OR 1.647; *p* = 0.191). 3. Intestinal gas volume: Intestinal gas volume was the only significant predictor (b = 0.001; OR 1.001 (95% CI [1.000–1.002]; *p* = 0.021) of the variables examined. An increased intestinal gas volume leads to a small but significant increase in the likelihood of a lethal outcome from resuscitation.

**Table 5:** Area Under the Curve.

				95% Confidence Interval	
	Area	Standard Error	Sig.	Lower Value	Upper Value
Stomach Gas	0.574	0.047	0.112	0.483	0.665
Intestinal Gas	0.671	0.045	0.000	0.583	0.760
Gastrointestinal gas	0.650	0.046	0.001	0.560	0.740

**Table 6:** Hemodynamic and pulmonary effects of abdominal insufflation.

Pulmonal Effects	Cardiac Effects
Reduced tidal and residual volume as well as compliance	Reduced contractility
Increased atelectasis	Reduced cardiac output
Increased shunt volume	Reduced diastolic filling
Increased pulmonary artery pressure	Compression vena cava inferior

## Data Availability

The participants in this study were not able to provide written consent for the publication of their original data; so, due to the sensitive nature of this research, no supportive data other than the presented results are available.
